# Inter-Protocol Interference Impact of LoRaWAN on IEEE 802.11ah in a Simulation Environment

**DOI:** 10.3390/s25226924

**Published:** 2025-11-13

**Authors:** Mateo Tito-Lara, Mauricio Domínguez-Limaico, Edgar Maya-Olalla, Fabián Cuzme-Rodríguez

**Affiliations:** Facultad de Ingeniería en Ciencias Aplicadas, Universidad Técnica del Norte, Av. 17 de Julio 5-21 y General José María Córdova, Ibarra 100105, Ecuador; matitol@utn.edu.ec (M.T.-L.); hmdominguez@utn.edu.ec (M.D.-L.); fgcuzme@utn.edu.ec (F.C.-R.)

**Keywords:** IEEE 802.11ah, LoRaWAN, NS-3, interference, throughput, SINR

## Abstract

The spectral coexistence of LPWAN technologies, such as IEEE 802.11ah and LoRaWAN, in the sub-GHz band presents significant challenges for the performance of dense IoT networks. This study analyzes the impact of LoRaWAN interference on IEEE 802.11ah using an NS-3-based simulation environment. To this end, both technologies were integrated within a unified simulation framework, enabling the configuration of PHY and MAC parameters, as well as operating frequency bands consistent with real-world deployments in the US902–928 MHz ISM band and aligned with official standards. The evaluation focuses on fundamental performance metrics—throughput, total packet loss percentage (PPP), and signal-to-interference-plus-noise ratio (SINR)—under varying node densities and payload configurations. Across our sweep, moving from the lowest to the highest LoRa load (from 10 to 8000 LoRa nodes within the specified deployment radius), IEEE 802.11ah throughput decreases by up to 31%, and the packet loss percentage (PPP) increases by up to 79%. Furthermore, an SINR threshold was established as the criterion for packet loss under interference. Overall, this work provides a reproducible methodology for assessing inter-protocol coexistence in unlicensed sub-GHz bands, contributing quantitative evidence to the analysis and design of multi-protocol IoT networks in dense environments.

## 1. Introduction

The field of the Internet of Things (IoT) is expanding rapidly and reaching multiple domains—industry, the smart grid, and smart cities, among others. Several market studies project substantial short-term growth; for example, Cisco estimates that by 2030, there will be approximately 500 billion IoT devices connected to the Internet [1], implying the coexistence of multiple technologies within the same ecosystem. In this landscape, low-power wide-area networks (LPWANs) stand out; they typically operate below 1 GHz (sub-GHz) and, for the most part, in unlicensed bands.

This work focuses on two LPWAN technologies—LoRa and IEEE 802.11ah—for which operating conditions depend on regional regulatory frameworks. In Europe, several 863–870 MHz sub-bands impose duty-cycle (often ≤1%) restrictions that limit transmission time; by contrast, in the United States (US902–928 MHz) and in many Latin American countries, there is no general duty-cycle cap, and compliance is instead defined through limits on radiated power, channel bandwidth, dwell time, and frequency-hopping requirements [2]. These regulatory differences, combined with increasing node density, raise the likelihood of interference among simultaneously operating LPWAN technologies, thereby compromising performance when multiple networks share the same segment of the radio spectrum.

In this context, a practical need arises: to quantify, with operational and realistic metrics, the unidirectional impact of LoRa transmissions on IEEE 802.11ah. This work addresses that need through a unified NS-3 simulation framework that integrates both technologies and explicitly models cross-technology interference. The model detects and measures the temporal overlap between each LoRa frame and the 802.11ah reception window at the *PPDU* level, and incorporates that overlap into the SINR computation via a PPDU-level decision rule to determine packet corruption under spectral coexistence. Using this approach, we evaluate the impact of LoRa on 802.11ah with the throughput, packet loss percentage (PPP), and signal-to-interference-plus-noise ratio (SINR) as key indicators.

This study makes the following contributions: (i) a unified NS-3 simulation framework that integrates LoRa and IEEE 802.11ah with coherent PHY/MAC parameterization and operation in sub-GHz bands, explicitly including the US902–928 MHz band and the exchange of temporal and spectral descriptors to assess coexistence under realistic conditions; (ii) a PPDU-level interference accounting mechanism for LoRa-to-802.11ah that detects and measures temporal overlaps and incorporates them into the computation of the effective SINR; (iii) an SINR threshold decision rule as an operational criterion for PPDU loss, supported by PER simulations; and (iv) a quantitative evaluation of the impact on IEEE 802.11ah—throughput, PPP, and SINR—under variations in LoRa density, load, and topology, including a sensitivity check to the deployment radius (200 m and extended scenarios), which confirms that the trends persist as the coverage area increases.

The present study outlines the following contributions: Section 2 contextualizes the problem and situates our contribution with respect to the state of the art; Section 3 provides an overview of IEEE 802.11ah and LoRaWAN (PHY/MAC aspects and sub-GHz regulatory considerations); Section 4 describes the integration in NS-3 and the key simulator modifications; Section 5 presents the experimental design and performance results (throughput, PPP, and SINR); and, finally, Section 6 synthesizes the main findings and outlines directions for future work.

## 2. Related Study

Several studies have explored the performance and interference characteristics of LPWAN technologies, particularly in unlicensed shared spectrum environments. A key study by Orfanidis et al. [3,4,5,6] investigates cross-technology interference (CTI) between LoRa and IEEE 802.15.4g networks operating in the 868 MHz band. This work experimentally analyzes the interference between LoRa and IEEE 802.15.4g, focusing on the packet reception rate (PRR) as the principal metric. The authors find that LoRa maintains a high PRR even in the presence of IEEE 802.15.4g interferers up to 16 dB stronger at higher spreading factors (SF9+). In contrast, IEEE 802.15.4g experiences a significant decrease in the PRR when LoRa’s bandwidth overlaps its channels, primarily due to the fundamental differences between LoRa’s chirp spread spectrum (CSS) and IEEE 802.15.4g’s Gaussian Frequency Shift Keying (GFSK). These findings are particularly relevant to our work, as they provide a foundation for understanding LoRa’s ability to maintain reliable communications under interference in the sub-GHz spectrum. We extend this baseline by examining the interaction between LoRaWAN and IEEE 802.11ah in a simulated environment. Unlike Orfanidis et al., who focus on experimental measurements involving IEEE 802.15.4g, our study utilizes simulations to model the impact of the higher data rates and channel access mechanisms of IEEE 802.11ah on LoRaWAN in dense scenarios, thereby offering a complementary perspective on cross-technology interference in IoT deployments.

While there is a precedent for a study comparing IEEE 802.15.4 and IEEE 802.11ah technologies [7] using metrics such as association time, throughput, and point-to-point delay, to date, no investigations have been conducted that evaluate the impact of interference and spectral coexistence between LoRaWAN and IEEE 802.11ah in dense simulation scenarios.

In addition, recent advances in wireless communication underscore the critical need for energy-efficient and reliable modulation schemes tailored to the diverse demands of Internet of Things (IoT) applications. Beyond conventional techniques, innovative approaches such as chaos-based modulation and spread-spectrum technologies have emerged as promising solutions. Notably, the multi-carrier initial-condition-index-aided differential chaos shift keying (MC-ICI-DCSK) scheme enhances spectral and energy efficiency in multipath fading channels by leveraging initial condition indices to embed additional information bits, achieving a robust detection mechanism via cross-correlation values [8]. Similarly, advancements in LoRa’s chirp spread spectrum (CSS) modulation, including the multiple-slope-keying CSS (MSK-CSS) and multi-layer superposition modulation (MLSM) schemes, significantly boost data rates by introducing a modulation factor to control frequency slopes and superimposing multiple signal layers, respectively, while maintaining reliable bit error rate (BER) performance [9]. These innovations reflect a growing trend toward scalable, low-cost, and resilient modulation strategies, paving the way for next-generation IoT networks capable of supporting long-range, low-power communications.

## 3. Overview of IEEE 802.11ah and LoRaWAN

This section examines the main functions of the technologies involved, separating their individual characteristics according to the first two layers of the OSI model. For more detailed information, it is recommended to consult the official standards [10,11].

### 3.1. IEEE 802.11ah PHY Physical Layer

The development of IEEE 802.11ah is based on IEEE 802.11ac, which is reflected in the use of technologies such as beamforming (beam shaping to increase signal strength relative to noise and terminal position) and MIMO [12].

#### 3.1.1. Channel Allocations

**Figure 1:**
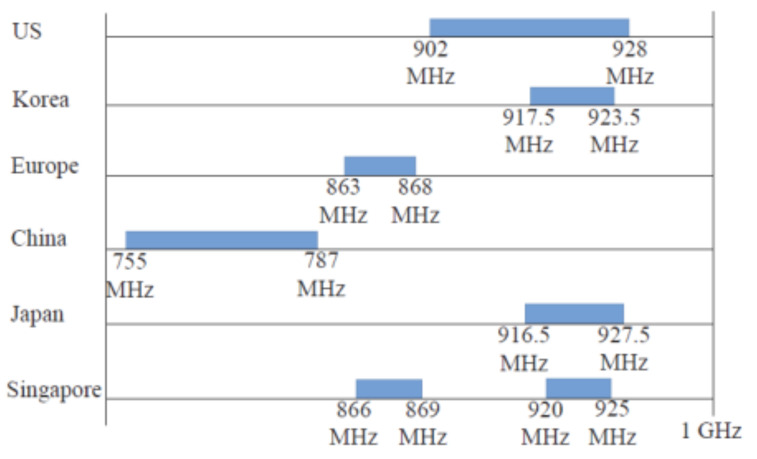
Channel allocation for various countries [13].

Channel allocations for IEEE 802.11ah vary by region. The data rate and modulation are determined by the MCS (Modulation and Coding Scheme) table. The sub-GHz bands differ depending on the regulatory bodies governing each country or continent, as shown in Figure 1. In the United States, the frequency band ranging from 902 to 928 MHz is available, totaling 26 MHz, according to FCC guidelines. This differs significantly from Europe, where the assigned frequency band falls between 863 and 868 MHz [13].

The most commonly used channel bandwidths are 1 MHz and 2 MHz, although some countries permit wider configurations of 4, 8, and 16 MHz. The PHY transmission uses an OFDM-based waveform composed of 32 or 64 tones/subcarriers spaced 31.25 kHz apart. The protocol enables modulations such as BPSK, QPSK, and QAM [12].

#### 3.1.2. Operating Modes

The design of the PHY layer can be classified based on transmission modes. In this work, the chosen mode is for a 1 MHz bandwidth channel (S1G_1M), which is suitable for applications requiring an extended operational range. In this mode, bandwidth and transmission speed are reduced to achieve longer distances. This is ideal for IoT applications that transmit data in bursts and do not require high data rates. Table 1 shows the MCS values for this configuration with one spatial stream ($N_{ss}$ = 1).

#### 3.1.3. Propagation Model

These are experimental mathematical models developed to characterize radio waves as a function of distance, frequency, or other conditions. The IEEE 802.11 working group developed urban-specific models for the TGah amendment (IEEE 802.11ah). The first, called “macro urban deployment”, considers an antenna 15 m above rooftop level, with path loss described by Equation (1) [14,15]:

(1) $$L\left(\mathrm{dB}\right)=8+37.6\log_{10}\left(d\right).$$

The second model, “urban peak/hotzone deployment,” assumes an antenna height of 2 m, appropriate for crowded areas. The model is given by

(2) $$L\left(\mathrm{dB}\right)=23.3+36.7\log_{10}\left(d\right).$$

#### 3.1.4. PHY PPDU Format

During transmission, the PSDU (PLCP Service Data Unit) is processed with the PHY preamble to form a PPDU (PLCP Protocol Data Unit). The structure for S1G_1M mode is described as follows, according to the IEEE Std 802.11ah-2016 [10]: The preamble includes a Short Training Field (STF) of 4 symbols, followed by a Long Training Field 1 (LTF1) of 4 symbols. This is succeeded by a Signal (SIG) field of 6 symbols, which contains control information. Subsequently, a sequence of fields from LTF2 to LTFnLTF is included, where each additional LTF field has 1 symbol per LTF, followed by the data field. The preamble also incorporates guard intervals (GI) and Long Training Symbols (LTS) distributed among the main components to ensure synchronization and error correction during transmission.

### 3.2. IEEE 802.11ah MAC Layer

The MAC layer is designed to support a large number of stations and minimize power consumption. IEEE 802.11ah includes features like the Restricted Access Window (RAW), which organizes channel access.

#### 3.2.1. Support for a Large Number of Stations

A standard IEEE 802.11 device supports up to 2007 identifiers. IEEE 802.11ah increases this to 8191 using a 13-bit Association ID (AID), enabling hierarchical grouping of stations.

#### 3.2.2. Restricted Access Window (RAW)

The RAW allows only specific devices to access the medium at scheduled intervals. These are divided into RAW slots, distributed using a round-robin scheme. The slot duration $D_{\mathrm{slot}}$ is calculated with

(3) $$D_{\mathrm{slot}}=500\;\mu s+C_{\mathrm{slot}}\cdot 120\;\mu s,$$

where $C_{\mathrm{slot}}$ depends on the Slot Definition Format Indication: if bit 0, then y=8; if bit 1, y=11. The number of RAW slots is $N_{\mathrm{RAW}}=14-y$, so the total window duration is

(4) $$D_{\mathrm{RAW}}=D_{\mathrm{slot}}\cdot N_{\mathrm{RAW}}.$$

### 3.3. PHY LoRa Layer

LoRaWAN uses a star-of-stars topology, where gateways relay messages between end devices (motes) and a central network server. Frequency allocations vary by region (see Table 2) [16].

#### 3.3.1. Spreading Factor (SF)

The spreading factor (SF) in LoRa (chirp spread spectrum, CSS) is defined as the number of bits per symbol. Consequently, each symbol comprises $2^{\mathrm{SF}}$ chips, and the bandwidth expansion factor is also $2^{\mathrm{SF}}$. For a configured bandwidth BW, the symbol rate is $R_{s}=\mathrm{BW}/2^{\mathrm{SF}}$, and the uncoded bit rate is $R_{b}=\mathrm{SF}\cdot \mathrm{BW}/2^{\mathrm{SF}}$ (with FEC, $R_{b}^{\mathrm{eff}}=R_{b}\cdot \mathrm{CR}$). Increasing SF therefore increases the symbol duration and reduces the symbol rate (for fixed BW), improving sensitivity and robustness to interference at the cost of lower throughput.

In the United States’ 902–928 MHz ISM band (US902–928), LoRa modulation defines five uplink data rates (DR0–DR4), employing spreading factors from SF7 to SF10 over 125 kHz channels, along with an additional configuration using SF8 over a 500 kHz channel. As summarized in Table 3, lower spreading factors combined with wider bandwidth yield higher data rates, whereas higher spreading factors provide extended coverage at the cost of reduced throughput, illustrating the inherent trade-off between transmission capacity and coverage range [17].

#### 3.3.2. PHY PPDU Format

**Figure 2:**
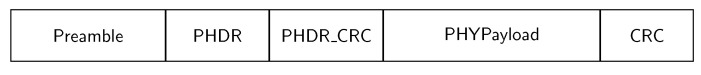
LoRa PHY PPDU format [16].

LoRa messages are transmitted using the Explicit Radio Packet mode, as illustrated in Figure 2. In this mode, the packet includes a physical-layer header (PHDR) and a cyclic redundancy check for the header (PHDR_CRC). To guarantee payload integrity, an additional CRC field is appended at the end of the frame. Both the PHDR and PHDR_CRC fields are automatically generated and processed by the end device transceiver (Tx/Rx).

#### 3.3.3. MAC LoRaWAN Layer

**Figure 3:**
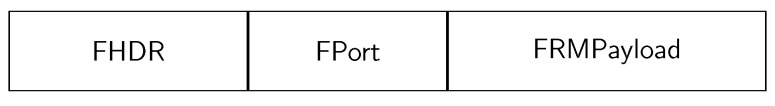
LoRa MAC Payload structure [18].

The MAC Payload structure, shown in Figure 3, results from the expansion of the MAC Payload field within a PHY Payload. It consists of a Frame Header (FHDR), followed by an optional Port field (FPort) and an optional Payload field (FRMPayload) [18]. This structure is defined by the LoRaWAN specification and provides the logical organization of data exchanged between end devices and the network server.

## 4. Implementation

This section presents the NS-3 simulation framework developed to assess the impact of LoRaWAN interference on IEEE 802.11ah under coexistence scenarios in the US 902–928 MHz ISM band. We focus on (i) the design rationale behind the architectural choices, (ii) how these choices support the study objectives, and (iii) the assumptions and constraints that bound the interpretation of the results.

### 4.1. Objectives and Justification of the Design

Our primary objective is to quantify the impact of inter-protocol interference (LoRaWAN → IEEE 802.11ah) on three metrics relevant to dense IoT deployments: throughput, packet loss percentage (PPP), and signal-to-interference-plus-noise ratio (SINR). Two design decisions were critical:

- Regulatory consistency (US902–928 MHz and regional variants): The coexistence evaluation reflects the channel plans used in the United States (US902–928 MHz) and in Latin America (e.g., Mexico 902–928 MHz, Brazil 902–907.5/915–928 MHz). We adapted the public NS-3 modules so that both technologies operate in these bands with consistent sub-bands and bandwidths, enabling fair comparisons under region-accurate allocations.
- Single, unified simulation environment: Integrating the IEEE 802.11ah and LoRaWAN models within one NS-3 workflow ensures consistent propagation, topology, and timing assumptions, enabling a fair comparison and controlled what-if analyses for density, payload, and timing overlap.

These choices prioritize methodological soundness over convenience: they minimize hidden inconsistencies (e.g., mismatched noise figures or disjoint timing bases) that could bias coexistence outcomes.

### 4.2. Module Adaptations

We relied on the community IEEE 802.11ah implementation by imec–IDLab [19] and the LoRa/LoRaWAN module by SignetLab–DEI [20]. Both were examined to identify gaps with respect to US operation and dense-coexistence testing. We implemented targeted adaptations to achieve the following:

1. Enable US 902–928 MHz operation in LoRaWAN: Sub-band definitions and downlink window frequencies were configured to match US channel plans (uplink channels of 125 kHz bandwidth), ensuring that LoRa transmissions occupy realistic center frequencies and bandwidths.
2. Harmonize PHY/MAC parameters in IEEE 802.11ah: We selected a 1–2 MHz channeling compatible with extended-range IoT use and adopted MCS 2 (QPSK) to reflect low-to-moderate data rates typical of sensor networks (see Table 1).
3. Unify timing and logging: LoRaWAN transmission descriptors (start/end times, center frequency, TX power, and node positions) are exported per iteration and ingested by the IEEE 802.11ah PHY to compute temporal/frequency overlap and interference consistently during reception.

### 4.3. Development of the Interference Accounting Model

With both modules operating in the US region, the LoRa module was modified to generate, at each simulation iteration, a descriptor file including the start and end times of every LoRaWAN transmission, station positions, transmission power, and the exact sub-band frequency used. These data are then imported into the IEEE 802.11ah simulation via the EndReceive routine of the PHY, which validates and finalizes reception. To assess whether any exported LoRa transmissions interfere with IEEE 802.11ah, the system first identifies the presence of partial, complete, or null temporal overlaps and computes their duration.

#### Temporal Overlap Detection

The system determines whether LoRa transmissions interfere with IEEE 802.11ah by checking the temporal intersection between each LoRa packet and the 802.11ah reception window. The detection routine—shown in Algorithm 1—computes the exact overlap duration. For reproducibility, the algorithm preserves the case structure of our implementation while remaining equivalent to the canonical interval-intersection formulation.

For completeness, the temporal overlap can be expressed in closed form via indicator functions $i_{1}\left(t\right)=1\left\{st_{1}<t<en_{1}\right\}$ and $i_{2}\left(t\right)=1\left\{st_{2}<t<en_{2}\right\}$:

(5) $$\mathrm{overlap}\;=\;\int_{-\infty }^{+\infty }i_{1}\left(t\right)\;i_{2}\left(t\right)\;dt\;=\;\max\;\left(0,\;\min\left(en_{1},en_{2}\right)-\max\left(st_{1},st_{2}\right)\right).$$

Equation (5) is algebraically equivalent to the procedure below, which mirrors our code structure for reproducibility.
**Algorithm 1** Temporal overlap detection and duration**Require:** *st*_1_, *en*_1_ (desired); *st*_2_, *en*_2_ (interferer)**Ensure:** *overlap*, *flag*  1: overlap ← max(0, min(*en*_1_, *en*_2_) − max(*st*_1_, *st*_2_))  2: *flag* ← (*overlap* > 0)  3: **return**
*overlap*


### 4.4. SINR Computation and Decision Rule

The SINR indicates the relationship between the signal, interference, and noise and is obtained in dB as

(6) $$10\cdot \log_{10}\left(\frac{\mathrm{Signal}}{\mathrm{Noise}+\mathrm{Interference}}\right).$$

If a temporal overlap is detected, the interfering LoRa power at the IEEE 802.11ah station is estimated via distance-dependent path loss:

(7) $$\mathrm{Path}\;\mathrm{Loss}\;\left(\mathrm{dB}\right)=10\cdot n_{PL}\cdot \log_{10}\;\left(\frac{d}{d_{ref}}\right)+\delta ,$$

where $n_{PL}=3$ (urban sub-GHz), *d* is the inter-node distance, and $d_{ref}=1$ m. The term δ accounts for the frequency correction relative to the 900 MHz reference and is defined as [15]

(8) $$\delta =21\cdot \log_{10}\;\left(\frac{f}{900\;\mathrm{MHz}}\right),$$

with a 30 dB reference loss added at 900 MHz [21]. The received LoRa power (dBm) is obtained by subtracting this attenuation from the LoRa transmit power and converting the result into watts to integrate the interference energy over the overlap duration. Multiple overlapping LoRa transmissions contribute additively to the total interference energy. Thermal noise is computed as

(9) $$\mathrm{NoiseT}\;\left(\mathrm{dBm}\right)=10\cdot \log_{10}\;\left(1000\cdot k\cdot T\cdot AB\right),$$

where $k=1.38\times 10^{-23}$ J/K is Boltzmann’s constant, T=200 K is the system temperature, and AB is the receiver bandwidth in hertz. The factor 1000 converts the noise power kTB from watts to milliwatts before applying the logarithmic transformation, thereby yielding the result in dBm.

#### Post-Decision Handling

Algorithm 2 applies the same decision rule consistently across the simulation batch, ensuring uniform handling when multiple interferers overlap with the same PPDU. For completeness, the symbols used are as follows: power (LoRa TX power, dBm), rxPowerdBm_lora (received interferer power, dBm), dBmToW(·) (power conversion), overlap (seconds), signalPowerW (desired-signal power, W), noisePowerW (from kTB via (9)), and δ (frequency correction in (7)). The cumulative interference energy aggregates contributions over all overlapping LoRa transmissions affecting the same IEEE 802.11ah PPDU.
**Algorithm 2** SINR calculation and threshold evaluation**Require:**   *positionRx*(x,y) position of the IEEE 802.11ah STA   *flag*Boolean indicating temporal overlap   *power*LoRa interferer TX power (dBm)   *signalPowerW*desired-signal power (W)   *overlap*overlap duration (s)   *position*(x,y) position of the interfering STA   *m*_*ref erenceLoss*=30, *m*_*exponent*=3, *m*_*referenceDistance*=1, *m*_ *frequency*=900e6**Ensure:**      SINR_dB_ and decision (drop/continue)  1:  if *flag* = false
**then**  2:    **return**                                      ▹ no temporal overlap ⇒ no interference contribution  3:  **end if**  4:  *distance* ← Euclidean(*positionRx*, *position*)  5:  *pathLossDB* ← 10·*m_exponent*·$\log_{10}\left(\frac{distance}{m\_referenceDistance}\right)+\delta$                
▹δ is the frequency correction  6:  *rxc* ← *pathLossDB* + *m_referenceLoss*  7:  *rxPowerdBm_lora* ← *power* − *rxc*  8:  *interfPowerW_lora* ← dBmToW(*rxPowerdBm_lora*)  9:  *interfEnergy* ← *interfPowerW_lora* · *overlap* 10: *cumulativeInterfEnergy* ← *cumulativeInterfEnergy* + *interfEnergy*  11: *signalEnergy* ← *signalPowerW* · *overlap*  12:  *thermalNoiseEnergy* ← *noisePowerW* · *overlap*         ▹*noisePowerW* from *kTB*  13:  *SINR* ← $\frac{signalEnergy}{cumulativeInterfEnergy+thermalNoiseEnergy}$
  14:  SINR_dB_ ← 10· log_10_*(SINR)*  15:  **if** SINR_dB_ < 16 **then**  16:         **drop** PPDU; NotifyRxDrop; set STA state → Error  17:  **else**  18:         **continue** reception (decapsulation)  19:  **end if** 20:  **return**


### 4.5. PER Target and BER Relationship

**Figure 4:**
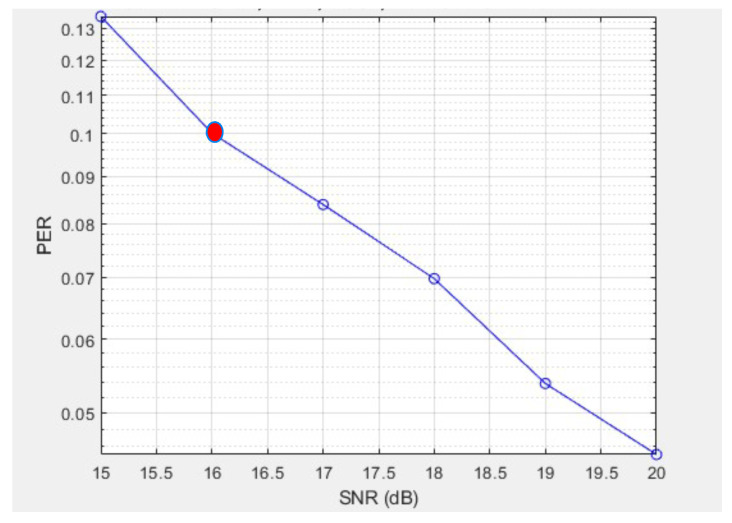
PER simulation for IEEE 802.11ah with SNR between 15 and 20 dB (MCS 2, payload 800 bits), confirming ≈0.1 at 16 dB.

The PER target is anchored in the IEEE 802.11ah specification (PER ≤ 10% for a 256-octet PSDU at −30 dBm input). PER and BER relate as

$$\mathrm{PER}=1-{\left(1-\mathrm{BER}\right)}^{L},$$

where *L* is the payload length in bits. For instance, assuming independent bit errors in an uncoded system, $\mathrm{BER}=10^{-3}$ and PER=0.1 yield L≈105 bits. However, IEEE 802.11ah MCS 2 includes convolutional coding (rate 3/4) and interleaving, which reduce the effective BER and support larger *L*, such as our simulation payload of 100 bytes (800 bits), achieving PER≈0.1 at 16 dB as shown in Figure 4 at the red dot. This aligns with the standard’s 256-octet (2048 bits) test case [10]. The literature BER curves for QPSK suggest SNR≈10 dB for $\mathrm{BER}=10^{-3}$ [22], while link-model studies offer complementary SINR mappings [23]. For clarity, Table 4 compares configurations.

#### 4.5.1. Discussion of the 16 dB SINR Decision Threshold

In the simulation we adopt a single 16 dB SINR threshold as a PPDU-level decision rule (the physical unit that includes the preamble and the PSDU). This value is grounded in (i) the IEEE 802.11ah requirement of PER ≤ 10% on the PSDU for a 256-octet payload, (ii) the selected operating point (MCS 2, QPSK), and (iii) complementary PER simulations in MATLAB indicating PER ≈0.1 around 16 dB under the conditions considered. Operating the decision at the PPDU-level is consistent with the PHY reception flow in NS-3: if the PPDU is not decoded, the PSDU cannot be recovered. This threshold relies on Equation (10) independent-error assumption post-coding; validity limitations in correlated fading are detailed in Section 4.5.

To properly frame this single-threshold approximation, we outline the operating assumptions and the factors that may affect its performance:

- Frequency selectivity and OFDM structure: The threshold aggregates interference at the PPDU-level and does not resolve per-subcarrier SINR. Under frequency-selective fading, coding and interleaving may tolerate deep fades over a subset of subcarriers, shifting the effective decision region.
- Coding gain and implementation margins: Different coding/interleaving schemes and receiver variants (e.g., LLR scaling, equalization) alter the PER–SINR mapping for the same MCS; therefore, a fixed threshold may be conservative or optimistic depending on conditions.
- Temporal dynamics and partial overlap: Asynchronous interferers may coincide with only a fraction of the PPDU. Although we integrate interference energy over the overlap duration, the exact timing (e.g., preamble/header vs. payload) can also modulate the impact.
- Variability of aggregate interference: Heterogeneous and bursty environments produce time-varying SINR margins. A fixed threshold captures an average operating point but not the full distribution of instantaneous margins.
- Capture effect and near–far situations: Very strong desired signals may be decoded at a lower SINR due to capture. The model does not explicitly represent this phenomenon beyond the energy-budget approximation.

#### 4.5.2. Comparison with Alternative Techniques

Higher-fidelity methods could be employed at the cost of increased complexity:

- Per-subcarrier modeling with OFDM demapping and block-level decoding (BER→PER).
- Effective SINR mappings (EESM/MIESM) that compress the subcarrier-wise SINR vector into a scalar for use with reference PER curves.
- Capture-aware models and packet-level interference cancellation.

We opt for the single-threshold approach to maximize computational viability, comparability, and reproducibility within a unified coexistence simulation framework while aligning with the standard’s PER target and a representative IoT MCS. This delimits the validity of the results: other configurations (e.g., different MCS) or more detailed models could shift the decision region. Our contribution focuses on revealing coexistence trends (PPP, throughput, SINR) under high density and temporal overlap; incorporating EESM/MIESM, per-subcarrier modeling, or explicit capture is identified as future work.

### 4.6. Assumptions, Constraints, and Impact on Results

We make the following assumptions to bound the problem and ensure comparability:

- Propagation model: Sub-GHz urban path loss with frequency correction; small-scale fading (e.g., Rayleigh or Nakagami-*m*) and per-subcarrier selectivity are not explicitly modeled, so interference is captured at the PPDU-level. Equation (10) assumes independent bit errors (e.g., AWGN channels), where packet success is the product of bit probabilities. In practical cases with a low SINR, deep fading, or correlated interference (e.g., LoRa CSS overlapping subcarriers), errors may correlate, invalidating the exact form as the joint probability differs from marginal products [24,25]. Our MATLAB simulations (Figure 4) incorporate full OFDM waveform, convolutional coding, and Viterbi decoding to mitigate correlations, but future work could include explicit correlated fading models.
- PHY mode selection: IEEE 802.11ah MCS 2 (QPSK) represents a low-to-moderate rate operating point typical of IoT traffic; results at other MCS levels would shift SINR tolerance and, consequently, PPP/throughput trends.
- Temporal and spectral criteria: Interference is accounted for only under temporal coincidence; non-overlap in frequency implies no contribution, mirroring practical coexistence where spectral separation mitigates interference.

The implications are as follows: (i) the reported PPP/throughput penalties reflect the chosen operating point and channel model; (ii) stronger coding/diversity could relax the 16 dB decision; and (iii) scenarios without temporal or spectral overlap are, by construction, benign in this framework.

### 4.7. Unified Model for Iteration Control

The IEEE 802.11ah simulations are conducted using the parameters summarized in Table 5. Most values may vary during execution, except those explicitly defined as input to the simulation executable. Given MCS 2, the payload length used (100 bytes) is not the maximum supported by the standard. LoRa simulations are configured with the parameters listed in Table 5, ensuring that both technologies are consistently modeled within the same framework.

The node positions of both technologies are generated using a uniform distribution over a 2D disk centered at the origin. We vary the deployment radius per scenario to study scaling effects: $R_{\mathrm{ah}}\in \left\{200,400\right\}\;m$ and $R_{\mathrm{LoRa}}\in \left\{200,500\right\}\;m$. In each run, the LoRa simulator exports the (x,y) coordinates, start/end times, transmit power, and center frequency of every transmission; these descriptors are ingested by the IEEE 802.11ah PHY. The receiver–interferer distance is computed from the MobilityModel positions using the Euclidean formula, and is then used in the path loss model and the previously detailed SINR calculation. To enable exact replication, we fix the NS-3 random seed at runtime --seed=1; all reported results correspond to a single run per scenario with that seed.

### 4.8. Automation Tool

**Figure 5:**
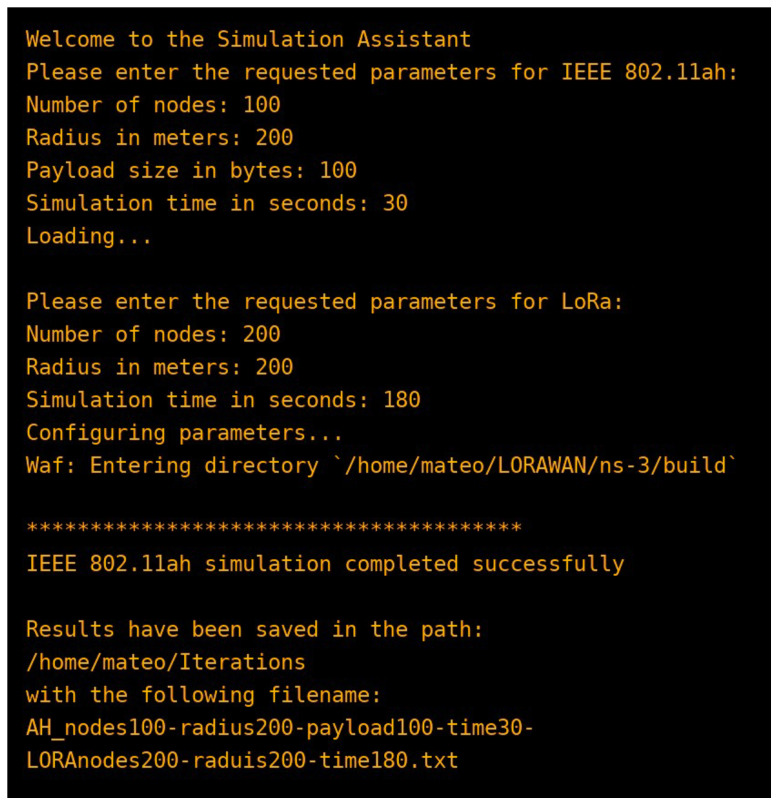
The interface of the executable tool.

To orchestrate the end-to-end process, an executable was developed to accept per-technology inputs, configure modules, run batch simulations, and consolidate outputs (see Figure 5). This tool assists reproducibility but does not alter the scientific assumptions above.

## 5. Evaluation

This section presents the results obtained from simulations carried out using modified modules for both technologies. The performance of the IEEE 802.11ah network under LoRa interference was assessed through three key metrics: throughput, the packet loss percentage (PPP), and the signal-to-interference-plus-noise ratio (SINR). These metrics are recognized as Key Performance Indicators (KPIs) in the ANSI C63.27-2017 standard (American National Standard for Evaluation of Wireless Coexistence) [26]. In addition, to provide deeper insights into inter-protocol interference, a dedicated subsection includes a theoretical and mathematical analysis of the phenomenon.

### 5.1. Theoretical and Mathematical Analysis

To characterize the behavior of IEEE 802.11ah and LoRa signals under interference conditions, a representative simulation iteration with 100 IEEE 802.11ah nodes and 10 LoRa nodes was selected. This configuration allowed the identification of two overlapping scenarios for detailed evaluation.

**Figure 6:**
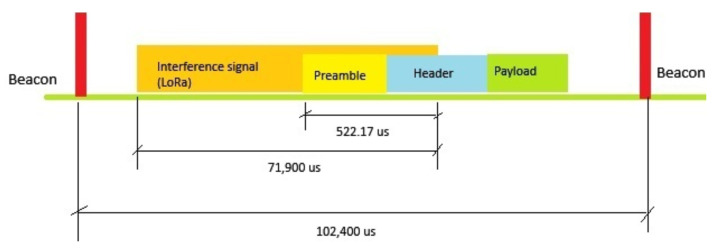
Partial overlap: the LoRa interferer corrupts the IEEE 802.11ah PPDU preamble (320 µs) and part of the header (within the total overlap of 522.17 µs).

The first scenario corresponds to a partial overlap. As illustrated in Figure 6, the LoRa transmission interferes with the IEEE 802.11ah signal for a total of 522.17 µs, fully disrupting the PPDU preamble (320 µs) and partially corrupting the header (240 µs).

**Figure 7:**
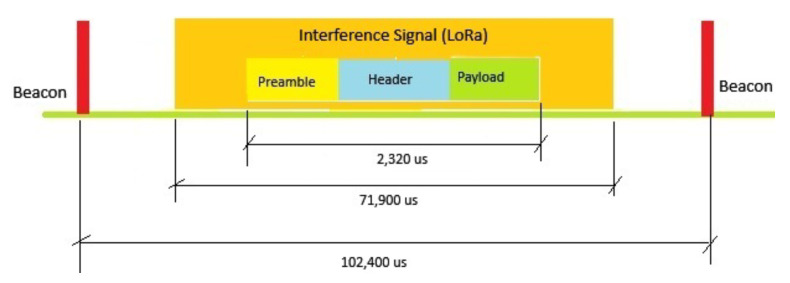
Complete overlap: the LoRa interferer overlaps the entire IEEE 802.11ah PPDU (2320 µs), corrupting the preamble, header, and payload.

In the second scenario, a complete overlap was identified. As shown in Figure 7, the entire IEEE 802.11ah PPDU duration of 2320 µs is corrupted by LoRa interference—preamble, header, and payload—preventing successful decoding.

**Figure 8:**
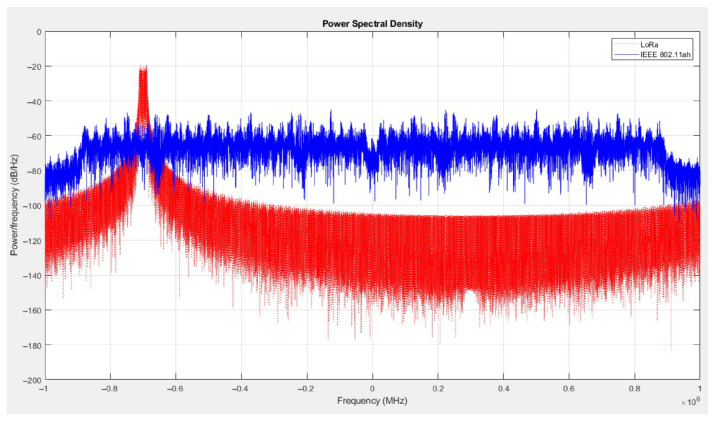
Power Spectral Density (PSD) of IEEE 802.11ah (blue) and LoRa (red). With 125 kHz LoRa bandwidth and 31.25 kHz 802.11ah subcarrier spacing, one LoRa channel can span up to four subcarriers.

To explicitly link spectral overlap to the physical-layer degradation observed in the metrics (lower throughput, higher PPP, and reduced SINR), we analyzed the spectral occupancy of both technologies via a Power Spectral Density (PSD) evaluation. IQ samples were generated in MATLAB^®^ 2023a: the LoRa transmission/reception followed the methodology in ref. [27], and the IEEE 802.11ah waveforms were produced with the WLAN Toolbox. Figure 8 compares the PSDs of IEEE 802.11ah (blue) and LoRa (red). Given that the evaluated IEEE 802.11ah channel is 2 MHz wide and its subcarrier spacing is 31.25 kHz, a 125 kHz LoRa signal spans a contiguous set of 802.11ah subcarriers—up to four, depending on frequency alignment. Critically, this set may include one or more pilot subcarriers used for channel estimation and carrier tracking; when those pilots are corrupted during temporal overlap, the receiver’s estimation degrades, reducing the effective SINR. This PSD-based mechanism is consistent with the observed trends—lower throughput and higher packet loss at higher LoRa densities—by directly linking spectral overlap to physical-layer impairment.

### 5.2. Metric Evolution

**Figure 9:**
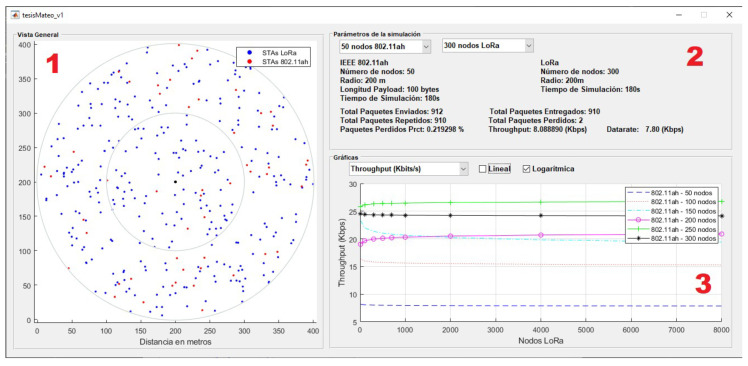
GUI developed for performance metric evaluation, divided into three sections: (1) spatial distribution of LoRaWAN and IEEE 802.11ah stations, (2) configuration parameters, and (3) combined metric plots for performance evaluation.

To facilitate the analysis of the collected results, a graphical user interface (GUI) was developed in MATLAB^®^. As shown in Figure 9, the GUI is organized into three sections: the first displays the spatial distribution of stations for both technologies in each simulation iteration; the second presents the configuration parameters for the selected variation, along with the main simulation results; and the third section includes combined metric plots for performance evaluation.

In consideration of the aforementioned factors and employing the parameters enumerated in Algorithms 1 and 2, a total of 40 individual simulations were conducted, varying the number of LoRa stations according to the number of IEEE 802.11ah stations. The resulting metrics are analyzed below.

### 5.3. Experimental Design and Reproducibility

We evaluated a grid of configurations by sweeping the number of IEEE 802.11ah stations and the number of LoRa stations:

$$N_{\mathrm{ah}}\in \left\{50,\;100,\;150,\;200\right\},\;N_{\mathrm{LoRa}}\in \left[10,8000\right]\cap \left\{10,100,300,500,700,1000,\ldots ,8000\right\}.$$

This yields 4×10=40 distinct configuration points.

Each configuration was executed once (R=1) with a fixed RNG seed and identical PHY/MAC settings across the sweep. Consequently, the figures report the single-run outcome for each configuration (no averaging across repetitions and no confidence intervals). This choice keeps the full coexistence sweep tractable and reproducible. A multi-seed repetition study is identified as future work to quantify run-to-run variability with confidence bounds.

### 5.4. Throughput

The throughput quantifies the amount of useful information transmitted per unit of time [27]. In the IEEE 802.11ah simulations, the throughput was computed using Equation (10):

(10) $$\mathrm{Throughput}\;\left(\mathrm{bps}\right)=\frac{(\mathrm{SuccessfulPkts}+\mathrm{RespondedPkts})\cdot \mathrm{PayloadLength}\cdot 8}{\mathrm{SimulationTime}}.$$

**Figure 10:**
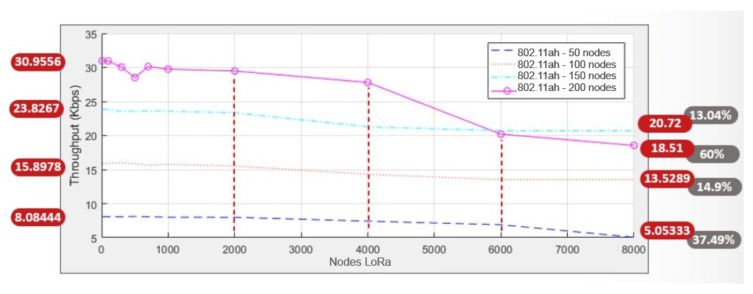
Combined throughput graph.

Figure 10 shows the throughput results across all simulations. A clear decreasing trend is observed as the number of LoRa nodes increases, with the degradation becoming more pronounced in scenarios with higher IEEE 802.11ah node density. Specifically, while the throughput remains relatively stable but low with 50 IEEE 802.11ah nodes, it starts at approximately 30 kbps with 200 nodes and decreases by more than 37% as the number of LoRa nodes approaches 8000. These results highlight that inter-protocol interference has a more severe impact in dense deployments, where both IEEE 802.11ah and LoRaWAN contend for the same sub-GHz spectrum resources.

### 5.5. Total Packet Loss

Total packet loss is computed as the fraction of data packets that are not successfully delivered. In the simulator, it is implemented as defined in (11):

(11) $$\mathrm{PPP}\left(\%\right)=100\cdot \left(1-\frac{\mathrm{totalDataPktsReceived}}{\mathrm{totalDataPktsSent}}\right).$$

**Figure 11:**
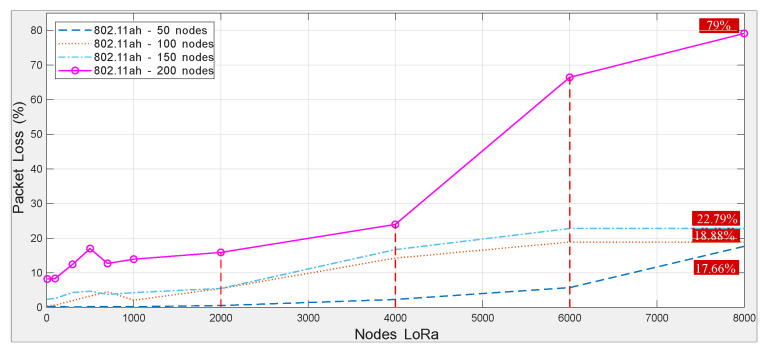
Combined graph of the packet loss percentage.

Figure 11 shows a clear positive correlation between the number of LoRa nodes and the packet loss percentage (PPP), which is the primary driver of the throughput degradation observed in Figure 10. In the absence of interference (i.e., zero LoRa nodes), the PPP ranges from 0.21% to 8.36% for 50 and 200 IEEE 802.11ah stations, respectively. As LoRa node density increases, the PPP rises substantially—up to 18.88–79%—reflecting growing contention and cross-technology interference in dense deployments.

### 5.6. Signal-to-Interference-Plus-Noise Ratio (SINR)

The SINR is a widely used indicator of channel quality and is defined as the ratio of the desired signal power to the sum of interference and thermal noise, as expressed in Equation (12).

(12) $$\mathrm{SINR}\left(\mathrm{dB}\right)=10\cdot \log_{10}\left(\frac{S}{I+N}\right)$$

**Figure 12:**
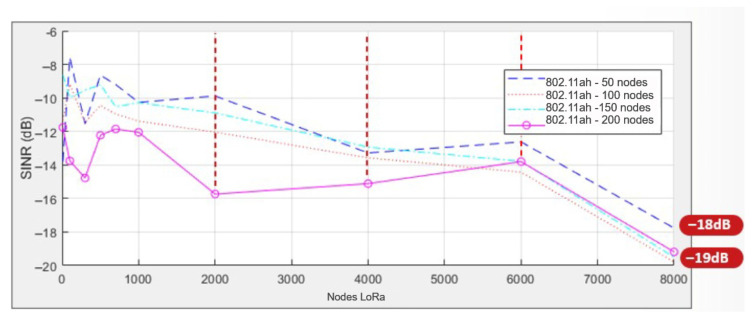
Combined SINR values across all simulation scenarios.

The SINR values reported in Figure 12 represent the average over all transmissions in which temporal overlap with LoRa interference was detected. As the network density increases, the average SINR decreases, primarily due to the cumulative effect of multiple LoRa interference sources.

### 5.7. Verification of Sensitivity Within a 500 m Radius

**Figure 13:**
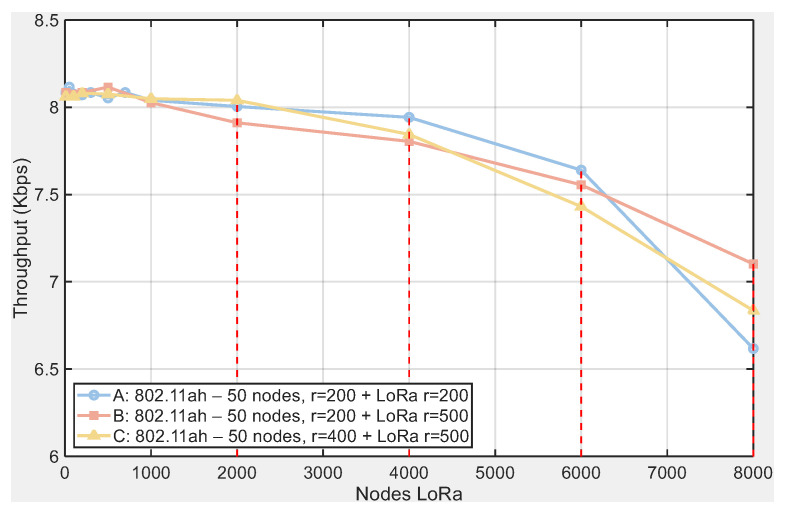
Throughput vs. LoRa nodes for three topologies: (A) 802.11ah 50 nodes r=200 m + LoRa r=200 m, (B) 802.11ah 50 nodes r=200 m + LoRa r=500 m, and (C) 802.11ah 50 nodes r=400 m + LoRa r=500 m.

**Figure 14:**
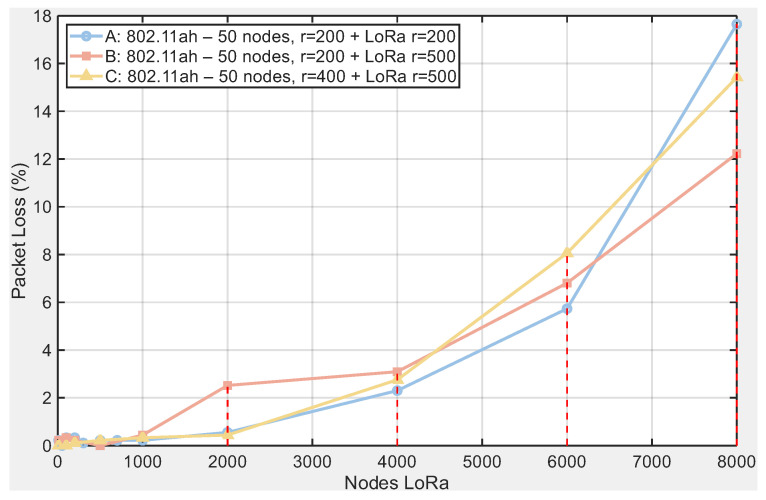
Packet loss percentage vs. LoRa nodes for the same topologies as Figure 13.

To verify the robustness of our conclusions with respect to topology size, we added scenarios with larger deployment radii. We evaluated three configurations while keeping 50 IEEE 802.11ah nodes and varying the deployment radius: (A) 802.11ah r=200 m + LoRa r=200 m; (B) 802.11ah r=200 m + LoRa r=500 m; and (C) 802.11ah r=400 m + LoRa r=500 m. The corresponding curves are shown in Figure 13 (throughput) and Figure 14 (PPP).

The results confirm the pattern observed in the baseline topology: throughput decreases and the PPP increases monotonically with LoRa density (see Figure 13 and Figure 14). Differences across radii remain moderate and do not alter the conclusions: at the highest load (8000 LoRa nodes), the final throughput lies in the range 6.62–7.10 kbps (A: 6.62; B: 7.10; C: 6.84), while the final PPP falls between 12.22 and 17.65% (A: 17.65%; B: 12.22%; C: 15.42%). At low and medium loads, the three configurations nearly overlap; only at high densities do differences of roughly 10–20% emerge depending on the radius combination. In summary, increasing the radius neither reverses the trends nor changes the main reading: coexistence with LoRa degrades IEEE 802.11ah performance increasingly with interferer density, including in 500 m scenarios.

## 6. Conclusions

This work presented a simulation-based assessment of the inter-protocol interference impact of LoRaWAN on IEEE 802.11ah in the sub-GHz band, implemented through modified NS-3 modules. Both technologies were integrated under realistic PHY/MAC configurations within a single, unified workflow, yielding a reproducible framework for coexistence analysis.

The primary contribution is a unified and reproducible NS-3 methodology that performs cross-technology interference accounting: LoRa transmissions are exported as per-packet descriptors (time, power, position, and center frequency) and ingested by the IEEE 802.11ah PHY to evaluate temporal/frequency overlap and compute the PPDU-level SINR consistently. Within this framework, we employ an operational 16 dB SINR decision rule—motivated by the IEEE 802.11ah PER target at a representative MCS and validated with MATLAB-based PER simulations—to determine packet success or loss.

The simulation results confirmed that the presence of LoRa transmissions significantly impacts IEEE 802.11ah network performance. Within the explored range—from the lowest to the highest LoRa load in the given deployment radius—IEEE 802.11ah throughput decreases by up to 31% and the packet loss percentage (PPP) increases by up to 79%. These findings highlight the vulnerability of IEEE 802.11ah to cross-technology interference in dense deployments.

The SINR analysis revealed a decreasing trend as network density increased, primarily due to the cumulative interference of multiple LoRa nodes. Nevertheless, the interference affects at most four subcarriers within an IEEE 802.11ah OFDM symbol, which explains why complete packet loss does not occur even at SINR values close to –20 dB.

To verify robustness with respect to topology size, we extended the analysis beyond the base radius and evaluated two additional configurations: (B) IEEE 802.11ah r=200 m with LoRa r=500 m and (C) IEEE 802.11ah r=400 m with LoRa r=500 m. The trends observed in the baseline scenario persist in these larger-radius settings, with only moderate quantitative differences at high LoRa densities (see Figure 13 and Figure 14). This confirms that the qualitative conclusions are stable under increased coverage radii.

Overall, this study provides quantitative evidence and a reproducible methodology for evaluating the coexistence of LPWAN technologies in an unlicensed spectrum. The results emphasize the need for interference-aware mechanisms and spectrum management strategies in dense IoT scenarios where IEEE 802.11ah and LoRaWAN are expected to coexist.

Future work will build on the proposed methodology and simulation framework, which have proven effective for modeling dense IoT coexistence scenarios with IEEE 802.11ah and LoRaWAN. Building on this foundation, we will study heterogeneous traffic patterns, adaptive channel access strategies, and dynamic spectrum management techniques oriented toward actions such as channel switching, coordinated RAW scheduling, fairness rules, and deferral. Evaluation will rely on concrete and interpretable metrics: channel-switching gain (increase in throughput and decrease in the PPP after the switch) and the success rate in finding non-overlapping channels; RAW efficiency (reduction in collisions, improvement in the PPP and throughput, and the proportion of traffic served within RAW windows); cross-technology fairness (Jain’s index computed over IEEE 802.11ah and LoRaWAN throughputs); and controller reaction time (latency from degradation detection to action and control stability, avoiding unnecessarily frequent configuration changes). In particular, the quantitative evidence presented here can support the design of coexistence mechanisms aligned with the objectives of IEEE 802.19.3 [28], addressing deployment conditions not yet fully explored by the standard, such as ultra-dense LPWAN environments and asymmetric traffic loads. Finally, extending this methodology to include centralized coordination schemes, adaptive interference-mitigation policies, and validation on software-defined radio (SDR) testbeds will strengthen its applicability to real-world IoT deployments.

## Figures and Tables

**Table 1:** S1G MCSs for a 1 MHz channel with $N_{ss}=1$.

MCS Idx	Mod	R	$N_{\mathrm{bpcs}}$	$N_{\mathrm{sd}}$	$N_{\mathrm{sp}}$	$N_{\mathrm{cbps}}$	$N_{\mathrm{dbps}}$	$N_{\mathrm{es}}$	Data_Rate (kbps)	
									8 μs GI	4 μs GI
0	BPSK	1/2	1	24	2	24	12	1	300.0	333.3
1	QPSK	1/2	2	24	2	48	24	1	600.0	666.7
2	QPSK	3/4	2	24	2	48	36	1	900.0	1000.0
3	16-QAM	1/2	4	24	2	96	48	1	1200.0	1333.3
4	16-QAM	3/4	4	24	2	96	72	1	1800.0	2000.0
5	64-QAM	2/3	6	24	2	144	96	1	2400.0	2666.7
6	64-QAM	3/4	6	24	2	144	108	1	2700.0	3000.0
7	64-QAM	5/6	6	24	2	144	120	1	3000.0	3333.3
8	256-QAM	3/4	8	24	2	192	144	1	3600.0	4000.0
9	256-QAM	5/6	8	24	2	192	160	1	4000.0	4444.4
10	BPSK	1/2 with 2× rep.	1	24	2	24	6	1	150.0	166.7

**Table 2:** Regulatory parameters for LoRaWAN operating frequency bands in the European Union and North America.

	Europe	North America
Frequency Band	863–870 MHz	902–928 MHz
Channels	10	64 + 8 + 8
Channel BW Up	125/250 kHz	125/500 kHz
Channel BW Dn	125 kHz	500 kHz
TX Power Up	+14 dBm	+20 dBm typ (+30 dBm allowed)
TX Power Dn	+14 dBm	+27 dBm
SF Up	7–12	7–10
Data Rate	250 bps–50 kbps	980 bps–21.9 kbps
Link Budget Up	155 dB	154 dB
Link Budget Dn	155 dB	157 dB

**Table 3:** LoRaWAN data rates and maximum payload sizes for the US902–928 MHz band. (*) Indicates downlink values.

DR	SF + BW	Up/Downlink	Bit Rate (bps)	MAC Payload (M)	(N)
0	SF10/125 kHz	Uplink	980	19	11
1	SF9/125 kHz	Uplink	1760	61	53
2	SF8/125 kHz	Uplink	3125	133	125
3	SF7/125 kHz	Uplink	5470	250	242
4	SF8/500 kHz	Uplink	12,500	250	242
8	SF12/500 kHz	Downlink	980	61 *	53 *
9	SF11/500 kHz	Downlink	1760	137 *	129 *
10	SF10/500 kHz	Downlink	3900	230 *	222 *
11	SF9/500 kHz	Downlink	7000	230 *	222 *
12	SF8/500 kHz	Downlink	12,500	230 *	222 *
13	SF7/500 kHz	Downlink	21,900	230 *	222 *

**Table 4:** Comparison of configurations for PER = 0.1.

Configuration	L (bits)	BER for PER = 0.1	Source
Uncoded example (independent errors)	∼105	$10^{-3}$	Equation (10), illustrative
Simulations (MCS 2, 100 bytes payload)	800	∼1.32 $\times 10^{-4}$	MATLAB 2023a (Figure 4) with coding/interleaving
Standard IEEE 802.11ah (256-octet PSDU)	2048	∼5.14 $\times 10^{-5}$	Ref. [10], Section 23.3.17.4

**Table 5:** Unified configuration parameters for IEEE 802.11ah and LoRa simulations. Payload length 100 bytes (800 bits) for IoT relevance, consistent with PER target via coding gain, as clarified in Equation (10) discussion.

IEEE 802.11ah		LoRa	
**Parameter**	**Value**	**Parameter**	**Value**
**PHY layer**			
TX Power (dBm)	0	TX Power (dBm)	14
TX/RX Gain (dB)	0	TX/RX Gain (dB)	0
Noise Figure (dB)	6.8	Noise Figure (dB)	6.8
Coding	BCC	Coding	—
Modulation	QPSK	Modulation	LoRa (CSS)
Channel/PHY BW	2 MHz	Channel/PHY BW	125 kHz
Operating Band	902–928 MHz	Operating Band	902–928 MHz
Sub-bands	—	Sub-bands (MHz)	902.2–903.7; 903.9–905.3; 905.5–906.9
Propagation Model	Macro Urban	Propagation Model	Long-distance
Error Rate Model	YansErrorRate	Error Rate Model	—
**MAC layer**			
DIFS Duration (µs)	264	Preamble Length (bytes)	10
SIFS Duration (µs)	160	SIFS/DIFS	—
PPDU Preamble (µs)	160,320	LoRa Preamble Time	—
PPDU Header (µs)	80,240	Header Time	—
Beacon Interval (µs)	102,400	Beaconing	—
TX Queue Size (pkts)	10	TX Queue Size	—
Transmission Interval (s)	1	Transmission Interval (s)	1
Station/STA Distribution	Random	Station Distribution	Random
**Performance/topology**			
Wi-Fi Mode/DR	MCS 2	DR/SF	SF 7–12
Max PER (%)	10	Max PER (%)	—
Payload Length (bytes)	100	Payload Length (bytes)	10–30
Topology Radius (m)	200	Topology Radius (m)	200–800
Number of Nodes	50–300	Number of Nodes	10–8000

## Data Availability

The data is available upon request.

## References

[1] CISCO (2020). Cisco IoT. https://www.miralishahidi.ir/resources/at-a-glance-c45-731471.pdf.

[2] Castells-Rufas D., Galin-Pons A., Carrabina J. (2018). The regulation of unlicensed sub-GHz bands: Are stronger restrictions required for LPWAN-based IoT success?. arXiv.

[3] Orfanidis C., Feeney L.M., Jacobsson M., Gunningberg P. Investigating interference between LoRa and IEEE 802.15.4g networks. Proceedings of the 2017 IEEE 13th International Conference on Wireless and Mobile Computing, Networking and Communications (WiMob).

[4] Feeney L.M., Orfanidis C., Jacobsson M., Gunningberg P. Preliminary results on LoRaWAN and IEEE 802.15.4-SUN interference. Proceedings of the 16th ACM Conference on Embedded Networked Sensor Systems (SenSys ’18).

[5] Orfanidis C., Feeney L.M., Jacobsson M. Measuring PHY layer interactions between LoRa and IEEE 802.15.4g networks. Proceedings of the 2017 IFIP Networking Conference (IFIP Networking) and Workshops.

[6] Orfanidis C., Feeney L.M., Jacobsson M., Gunningberg P. (2017). Improving LoRa/IEEE 802.15.4g Co-Existence.

[7] Ahmed N., Rahman H., Hussain I. (2016). A Comparison of 802.11ah and 802.15.4 for IoT. https://www.researchgate.net/publication/305991052_A_comparison_of_80211ah_and_802154_for_IoT.

[8] Ma H., Tao Y., Fang Y., Chen P., Li Y. (2025). Multi-Carrier Initial-Condition-Index-aided DCSK Scheme: An Efficient Solution for Multipath Fading Channel. IEEE Trans. Veh. Technol..

[9] Luo J., Bai Y., Bai B., Chen C., Wen W. A Multi-layer Superposition Modulation Scheme to Improve the Data Rate for IoT Communications. Proceedings of the 2023 IEEE/CIC International Conference on Communications in China (ICCC Workshops).

[10] (2017). IEEE Standard for Information Technology–Telecommunications and Information Exchange Between Systems-Local and Metropolitan Area Networks–Specific Requirements-Part 11: Wireless LAN Medium Access Control (MAC) and Physical Layer (PHY) Specifications Amendment 2: Sub 1 GHz License Exempt Operation.

[11] LoRa Alliance Technical Specifications. https://resources.lora-alliance.org/technical-specifications.

[12] Adame T., Bel A., Bellalta B., Barcelo J., Oliver M. (2014). IEEE 802.11AH: The WiFi approach for M2M communications. IEEE Wirel. Commun..

[13] Keysight Technologies (2017). RFMW. http://rfmw.em.keysight.com/wireless/helpfiles/n7617/Content/Main/802.11ah%20Channelization.htm.

[14] Erceg V., Schumacher L., Kyritsi P., Molisch A., Baum D.S., Gorokhov A.Y., Oestges C., Li Q., Yu K., Tal N. (2004). TGn Channel Models. IEEE 802.11-03/940r4. https://mentor.ieee.org/802.11/dcn/03/11-03-0940-04-000n-tgn-channel-models.doc.

[15] Porat R., Yong S.K., Doppler K. (2011). TGah Channel Model–Proposed Text. IEEE 802.11-11/0968r4. https://mentor.ieee.org/802.11/dcn/11/11-11-0968-04-00ah-channel-model-text.docx.

[16] LoRa Alliance (2015). LoRa Alliance. https://lora-alliance.org/wp-content/uploads/2020/11/what-is-lorawan.pdf.

[17] Semtech (2015). LoRa Developers. https://www.semtech.com/lora.

[18] LoRa Alliance Technical Committee (2017). LoRaWAN 1.1 Specification.

[19] Tian L., Sljivo A., Santi S., Poorter E.D., Hoebeke J., Famaey J. Extension of the IEEE 802.11ah ns-3 simulation module. Proceedings of the 2018 Workshop on ns-3 (WNS3).

[20] SignetLab-DEI LoRaWAN Module for ns-3. https://github.com/signetlabdei/lorawan.

[21] Abdul Rahman T., Abdul Aziz O. (2014). Modelling the impact of operating frequencies on path loss and shadowing along multi-floor stairwell for 0.7 GHz–2.5 GHz range. Prog. Electromagn. Res. M.

[22] Hervas M., Alsina-Pagès R.M., Pijoan J.L., Salvador M. (2014). Single-carrier frequency domain equalisation proposal for very long haul HF radio links. Electron. Lett..

[23] Chen H. (2010). RLINK: A realistic simulation model of links in wireless sensor networks. J. Syst. Simul..

[24] Proakis J.G., Salehi M. (2008). Digital Communications.

[25] Simon M.K., Alouini M.-S. (2005). Digital Communication over Fading Channels.

[26] (2017). American National Standard for Evaluation of Wireless Coexistence.

[27] Al Homssi B., Dakic K., Maselli S. (2021). IoT Network Design using Open-Source LoRa Coverage Emulator. IEEE Access.

[28] (2021). IEEE Recommended Practice for Local and Metropolitan Area Networks–Part 19: Coexistence Methods for IEEE 802.11 and IEEE 802.15.4 Based Systems Operating in the Sub-1 GHz Frequency Bands.

